# The aging Canadian population and hospitalizations for acute myocardial infarction: projection to 2020

**DOI:** 10.1186/1471-2261-12-25

**Published:** 2012-04-02

**Authors:** Nigel SB Rawson, Rong Chu, Afisi S Ismaila, Jorge Alfonso Ross Terres

**Affiliations:** 1 Medical Affairs, GlaxoSmithKline Inc, 7333 Mississauga Road, Mississauga L5N 6L4, ON, Canada; 2 Department of Clinical Epidemiology and Biostatistics, McMaster University, Hamilton, ON, Canada

## Abstract

**Background:**

The risk of experiencing an acute myocardial infarction (AMI) increases with age and Canada's population is aging. The objective of this analysis was to examine trends in the AMI hospitalization rate in Canada between 2002 and 2009 and to estimate the potential increase in the number of AMI hospitalizations over the next decade.

**Methods:**

Aggregated data on annual AMI hospitalizations were obtained from the Canadian Institute for Health Information for all provinces and territories, except Quebec, for 2002/03 and 2009/10. Using these data in a Poisson regression model to control for age, gender and year, the rate of AMI hospitalizations was extrapolated between 2010 and 2020. The extrapolated rate and Statistics Canada population projections were used to estimate the number of AMI hospitalizations in 2020.

**Results:**

The rates of AMI hospitalizations by gender and age group showed a decrease between 2002 and 2009 in patients aged ≥ 65 years and relatively stable rates in those aged < 64 years in both males and females. However, the total number of AMI hospitalizations in Canada (excluding Quebec) is projected to increase by 4667 from 51847 in 2009 to 56514 in 2020, a 9.0% increase. Inflating this number to account for the unavailable Quebec data results in an increase of approximately 6200 for the whole of Canada. This would amount to an additional cost of between $46 and $54 million and sensitivity analyses indicate that it could be between $36 and $65 million.

**Conclusions:**

Despite projected decreasing or stable rates of AMI hospitalization, the number of hospitalizations is expected to increase substantially as a result of the aging of the Canadian population. The cost of these hospitalizations will be substantial. An increase of this extent in the number of AMI hospitalizations and the ensuing costs would significantly impact the already over-stretched Canadian healthcare system.

## Background

Despite therapeutic advances in the treatment of acute myocardial infarction (AMI) over the past two decades, the condition continues to be a leading cause of death in older adults in developed countries. Since the risk of experiencing an AMI increases with age [1], changes in the occurrence of this disorder have a major impact on the overall health of a country's population and its healthcare system.

In Canada, the population is aging and the country is particularly impacted by the baby boom generation. Baby boomers constitute almost one-third of the Canadian population, a larger proportion than in the other three countries (Australia, New Zealand and the United States) that experienced the baby boom effect. From 2008 onward, the leading edge of the boomer generation (those born between 1947 and 1966 [2]) moved into their 60s, while its youngest members edged into their 40s. Aging baby boomers have the potential to stretch an already over-extended healthcare system over the next few decades.

In addition to the aging population, there is an unprecedented and growing number of young Canadian adults who are overweight or obese [3], women are entering their adult years with a higher risk for heart disease [4] and some of Canada's fastest growing ethno-cultural communities are predisposed to a heavier burden of risk factors for cardiovascular disease [5,6]. All these factors could potentially overwhelm the healthcare system with an entire new generation of patients [7].

The objective of this analysis was to examine trends in the hospitalization rate for AMI in Canada between 2002 and 2009 and to estimate the potential increase in the number of hospitalizations for AMI over the next decade.

## Methods

Aggregated data on annual hospitalizations with a most responsible (principal) diagnosis of AMI using International Classification of Diseases (ICD) codes (ICD-9 410 and ICD-10-CA I21 and I22) were obtained from the Discharge Abstract Database and Hospital Morbidity Database of the Canadian Institute for Health Information (CIHI) for nine of Canada's 10 provinces and all three of its territories for the fiscal years 2002/03 to 2009/10 inclusive [8]. The CIHI data record hospitalizations only and it is not possible to link hospitalizations for individual patients so that we could not link a re-admission for the same AMI episode or a new AMI with a previous one in the same patient. Information was only available for 2002/03-2009/10 and, since data from Quebec were unavailable for most of the time period, Quebec was excluded from the analysis.

The CIHI data were categorized into 10 age groups starting at < 45 years (since this is the age at which the risk of an AMI begins to increase) and ending at ≥ 85 years and these were re-grouped into four categories (< 50, 50-64, 65-74 and ≥ 75 years) for consistency with previous analyses [1,9,10]. Data were also obtained on the two most relevant revascularization procedures (percutaneous coronary intervention [PCI] and/or coronary artery bypass graft [CABG]) and the length of stay (LOS), categorized as 1-3 days, 4-6 days, 7-11 days or 12 + days by CIHI, associated with each hospitalization. The mean LOS for each gender/age group in each year was estimated using the midpoint of each of the first three categories and 14 days for the final one.

Using provincial and territorial mid-year population estimates obtained from Statistics Canada, annual rates of AMI hospitalization and associated rates of revascularization procedures were estimated for the four age groups stratified by gender. The rates were calculated by dividing the number of hospitalizations for each age-gender category by the corresponding population estimate and age and gender standardized to the 2002 population.

The annual rates of AMI hospitalization for 2002 to 2009 were analyzed using a log-linear Poisson regression model to control for age (four categories), gender and fiscal year (as a continuous variable ranging between zero and seven). The use of four age groups led to a larger number of hospitalization events within each age category and, subsequently, to a more parsimonious and stable model. Two-way interaction terms between age category, gender and fiscal year were also considered in the Poisson model to increase its flexibility. The annual population estimate in each age-gender category (eight in total) was included as an offset term to account for different size of the population at risk. The models were fitted on 64 observed data points (four age groups X two genders X eight years) using a quasi-likelihood approach to account for over-dispersion. The unit of analysis can be considered as person-year assuming all eight age-gender groups were at risk of being hospitalized for AMI throughout a year. An over-dispersion parameter, estimated by dividing the square root of the Pearson *χ*^2 ^value by the residual degrees of freedom, was introduced to reflect the greater variability in the observed data than expected from the Poisson distribution [11,12]. The model goodness-of-fit was examined using the deviance and Pearson *χ*^2 ^values. Final model selection was based on change in the Akaike information criterion, a measure of the relative goodness of fit of a statistical model, and the Bayesian information criterion.

The regression model was used to estimate the rate of AMI hospitalizations between 2010 and 2020. Statistics Canada has projected the national and provincial populations to 2036 based on the 2009 population and a series of assumptions which are "summarized into a few scenarios that are intended to be both plausible and relevant" so that they "offer a range of possibilities as to how the Canadian population will evolve in the future"[13]. The projections are produced by means of a micro-simulation method using a number of variables that reflect the ethno-cultural diversity of the Canadian population, with the basic geographic level being country's 33 census metropolitan areas. The projections are intended for a wide range of users including the federal government, provincial, territorial or municipal governments, research groups, academics, interest groups, international and non-governmental organizations, educational institutions and the general public.

The projections are based on six scenarios (Table 1) that use varying estimates of fertility, life expectancy, immigration and interprovincial migration. The most important of these is the first medium growth scenario (number 2 in Table 1), which Statistics Canada generally uses in its own analyses. In previous projections, the medium scenario has been found to be close to reality [13]. Using the population projections of scenario 2 and the AMI rate estimates, annual absolute numbers of AMI hospitalizations for the eight age-gender categories between 2010 and 2020 were estimated. Population projections from Statistics Canada's low and high scenarios (numbers 1 and 6 in Table 1) were used as a sensitivity assessment.

**Table 1:** The six scenarios used by Statistics Canada for population projection [13]

Scenario	Fertility	Life expectancy	Immigration	Interprovincial migration	
1	Low growth	Low	Low	Low	Historical trends (1981-2008)
2	Medium growth, historical trends (1981 to 2008)	Medium	Medium	Medium	Historical trends (1981-2008)
3	Medium growth, trends 2006 to 2008	Medium	Medium	Medium	2006 to 2008 trends
4	Medium growth, trends 1988 to 1996	Medium	Medium	Medium	1988 to 1996 trends
5	Medium growth, trends 2001 to 2006	Medium	Medium	Medium	2001 to 2006 trends
6	High growth	High	High	High	Historical trends (1981-2008)

Data from CIHI on estimates of the average cost of an AMI without and with a cardiac catheter in Canada [14] were used to estimate the impact of projected changes in the number of AMI hospitalizations.

The data on PCI and CABG were examined to assess what percentage of the hospitalizations included these procedures. Similarly, trends in LOS were reviewed.

## Results

**Figure 1:**
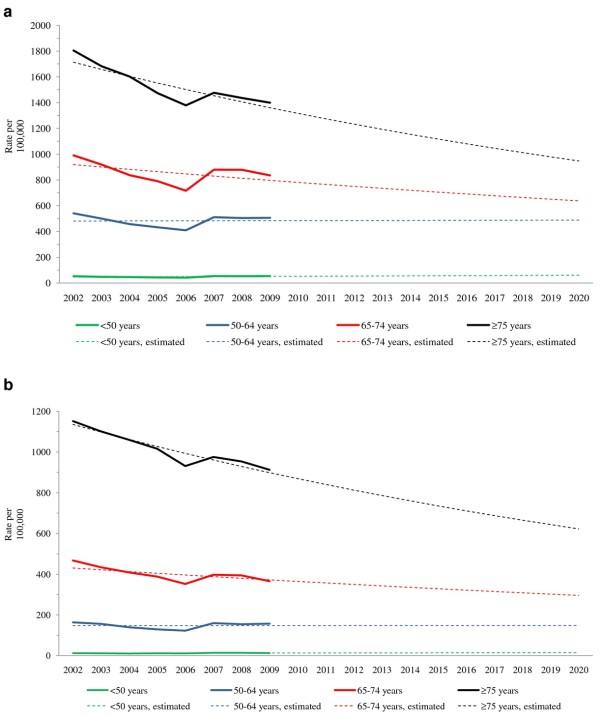
**a Actual and projected rate of AMI hospitalization in Canadian males by age group, 2002-2020**. AMI: Acute myocardial infarction. **b **Actual and projected rate of AMI hospitalization in Canadian females by age group, 2002-2020. AMI: Acute myocardial infarction.

The rates of AMI hospitalizations by gender and age group are shown in Figure 1a and 1b. In general, the rates show a decrease between 2002 and 2009 in the two oldest age groups (65-74 and ≥ 75 years) and a modest increase in the younger age groups (< 50 and 50-64 years) in both sexes.

The final Poisson regression model contained three main effects (age, gender and year) and three two-way interactions between the main effects (Appendix). This reflected the observed difference in the rate of AMI hospitalization between eight age by gender groups at baseline (year 2002) and allowed year to have different effects on the AMI rate among the groups. Using the Poisson regression model, the projections of the rates between 2010 and 2020 show stable estimates of the rate of AMI hospitalization in males and females aged < 65 years and decreasing rates in patients aged ≥ 65 years (Figure 1a and 1b), although the model was a better fit in females than males.

The estimated rates, together with the population projections from Statistics Canada, were used to project the number of AMI hospitalizations in Canada (excluding Quebec) to 2020. Table 2 shows the observed and predicted absolute numbers of AMI hospitalizations for males and females in 2009 and the projected numbers for 2020 for the low, medium and high Statistics Canada scenarios. Under the medium scenario, although AMI hospitalizations in the oldest age group are anticipated to decrease, the total number of AMI hospitalizations in Canada (excluding Quebec) is projected to increase by 4667 from 51847 in 2009 to 56514 in 2020, a 9.0% increase. The projected numbers under the low and high scenarios are 55538 and 57497, respectively, which represent increases of 3691 (7.1%) and 5650 (10.9%).

**Table 2:** Observed and estimated absolute numbers of AMI hospitalizations in 2009 and 2020 for Canada (excluding Quebec)

		2009		2020 projections for 3 population scenarios*					
		
Gender	Age group	Observed	Estimated	Low		Medium		High	
				N	%^†^	N	%^†^	N	%^†^
Male	< 50 years	4739	4541	5412	14.2	5639	19.0	5863	23.7
	50-64 years	12600	12053	14372	14.1	14528	15.3	14689	16.6
	65-74 years	7413	7073	9057	22.2	9167	23.7	9287	25.3
	≥ 75 years	9444	9182	8730	-7.6	8923	-5.5	9133	-3.3
	All ages	34196	32849	37571	9.9	38257	11.9	38972	14.0
Female	< 50 years	1102	1010	1307	18.6	1364	23.8	1421	28.9
	50-64 years	3988	3758	4425	11.0	4468	12.0	4511	13.1
	65-74 years	3531	3593	4536	28.5	4579	29.7	4618	30.8
	≥ 75 years	9030	8888	7699	-14.7	7846	-13.1	7875	-12.8
	All ages	17651	17249	17967	1.8	18257	3.4	18525	5.0
Total		51847	50098	55538	7.1	56514	9.0	57497	10.9

*AMI *Acute myocardial infarction

* Using Statistics Canada population projections

† Percentage change over the 2009 observed figure

**Figure 2:**
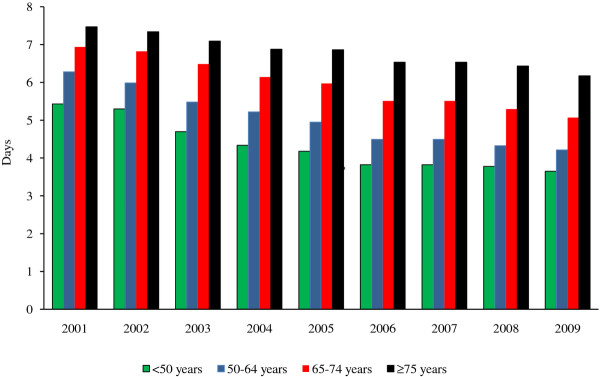
**Estimated mean length of hospital stay for AMI by age group, 2002-2009**. AMI: Acute myocardial infarction.

The estimated mean LOSs between 2002 and 2009 for males and females combined in each age group and each year are shown in Figure 2. The genders were combined because there was little difference between them, although the LOS for females was always slightly greater than that for males. The mean LOS declined in each age group by 17-33%, with decreases of 1.8, 2.1, 1.9 and 1.3 days in patients aged < 50, 50-64, 65-74 and ≥ 75 years, respectively.

**Figure 3:**
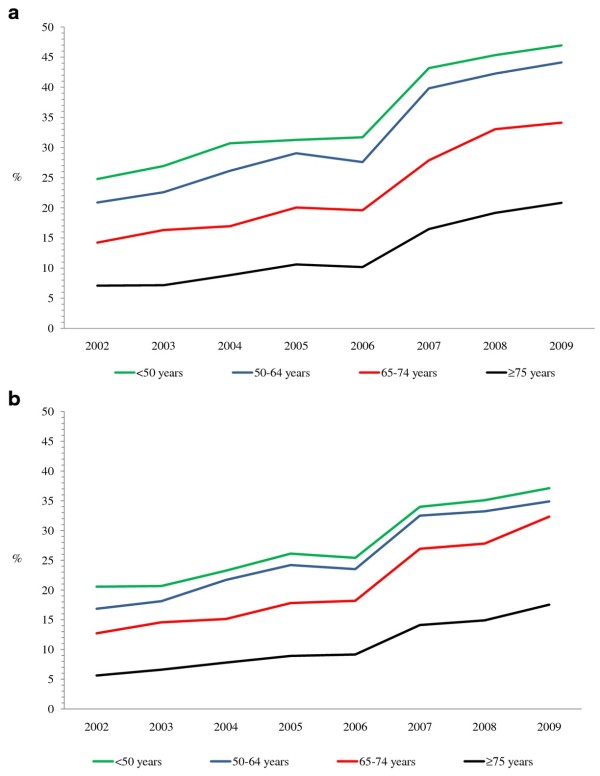
**a Percentage of AMI hospitalizations with a PCI in Canadian males by age group, 2002-2009**. AMI: Acute myocardial infarction. PCI: Percutaneous coronary intervention. b Percentage of AMI hospitalizations with a PCI in Canadian females by age group, 2002-2009. AMI: Acute myocardial infarction, PCI: Percutaneous coronary intervention.

**Figure 4:**
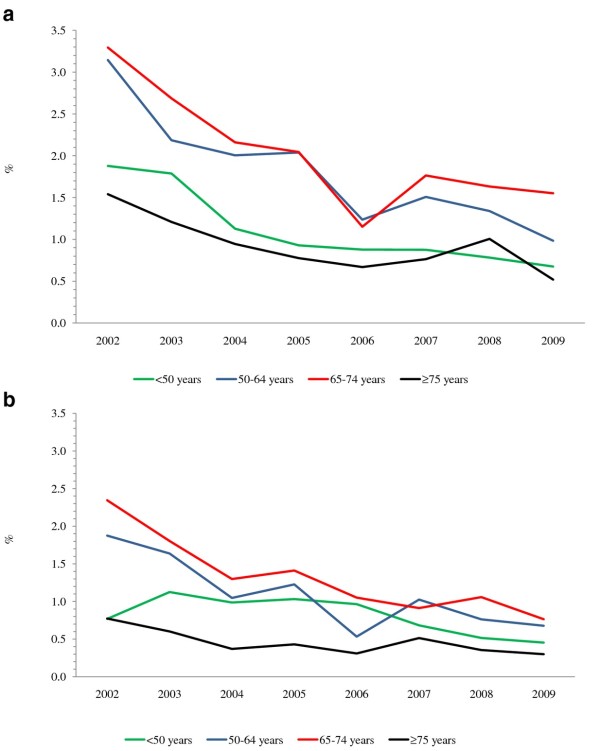
**a Percentage of AMI hospitalizations with a CABG in Canadian males by age group, 2002-2009**. AMI: Acute myocardial infarction. CABG: Coronary artery bypass graft. **b **Percentage of AMI hospitalizations with a CABG in Canadian females by age group, 2002-2009. AMI: Acute myocardial infarction. CABG: Coronary artery bypass graft.

The percentage of AMI hospitalizations with a PCI in Canada (excluding Quebec) increased between 2002 and 2009 in both males and females in each age group (Figure 3a and 3b). The increase was greatest in patients aged ≥ 75 years (three-fold), compared with approximately a two-fold increase in the other age groups. In contrast, the percentage of AMI hospitalizations with a CABG decreased in the eight-year period by more than half in both males and females from 2.5% and 1.3%, respectively, in 2002 to 0.9% and 0.5% in 2009 (Figure 4a and 4b).

## Discussion

Hospital discharge abstract databases are increasingly being used in health services research. AMI diagnostic codes in Canadian administrative databases have been validated using the hospital discharge data and chart audits [15-18]. For example, Austin *et al. *found a sensitivity of 89% and a specificity of 93% for patients admitted to coronary care units [17]. Administrative data have been used in surveillance of cardiovascular surveillance [1,9,10,19-21] and other conditions [22] with success.

The decreasing hospitalization rate for AMI in Canada has been described by others previously. In particular, in 2009, Tu and his colleagues reported a substantial decrease in hospitalization rates (standardized to the 1991 population) for AMI and other cardiovascular disorders from 1994 to 2004 using CIHI data for the whole of Canada [1]. Our study resulted in a rate for 2004 are quite similar to that of Tu *et al.*, despite not having data for Quebec.

Similarly, the increasing use of PCI and the declining use of CABG in AMI patients have also been previously reported. Ko *et al. *showed that, between 2001-03 and 2004-06, the rate of PCI use per 100,000 population in Ontario rose by 20%, while the rate of CABG use fell by 10% [23]. In the CIHI data, the rate of PCI in Canada (excluding Quebec) increased between 2002 and 2009 by 125% and the rate of CABG declined by over 60%. Thus, there has been a significant change in the type of care (PCI v. CABG) over recent years in Canada with PCI, which has been described as having a direct impact on patient survival. PCI remains a central therapy for patients with symptomatic coronary artery disease, particularly those with AMI, and has generated tremendous attention in the last decade with issues such as the risks and benefits of drug-eluting stents and anti-thrombotic therapies [24]. There are few representative data describing the contemporary patterns of care and outcome trends in patient with AMI and/or undergoing PCI. This is of particular importance because the process of updating clinical practice guidelines and quality metrics for AMI and PCI has accelerated.

The present analysis extends the previous work of Tu *et al. *[1] to a more recent year (2009) and attempts to project not only the rates, which our analysis suggests will continue to decline in those aged ≥ 65 years and remain relatively stable in those aged < 65 years, but also the numbers of AMI hospitalizations to 2020. Despite projected decreasing or stable rates of AMI hospitalization, the number of hospitalizations is expected to increase substantially as a result of the aging of the Canadian population.

Our model indicates an increase in Canada (excluding Quebec) of 4667 AMI hospitalizations by 2020, with a sensitivity range of 3691 to 5650. Quebec represents approximately 25% of the Canadian population, which suggests an increase of about 6200 for the whole of Canada (sensitivity range, 4900 to 7500). Using the CIHI estimate for the average cost of a myocardial infarction/shock/arrest without cardiac catheter in Canada in 2008/09 of $7412 [14], the additional cost for this increase in AMI hospitalizations would be approximately $46 million (sensitivity range, $36-$55 million). If one assumes that the majority of AMI hospitalizations (80%) will include a cardiac catheter for which the average cost in Canada in 2008/09 was $8984 [14], the additional cost for the projected increase in the number of AMI hospitalizations to 2020 would be approximately $54 million (sensitivity range, $42-$65 million). These figures do not take account of economic inflation, changes in AMI risk due to the epidemic in diabetes and obesity, or the fact that seniors generally have a longer LOS.

Attempting to incorporate changes in the use of PCI or CABG and in LOS with the projected trend in AMI hospitalizations was problematic. If one extrapolates the linear increase in the proportion of AMI hospitalizations with a PCI between 2002 and 2009, by 2020 almost all AMI hospitalizations would have a PCI. Projecting the proportion of AMI hospitalizations with a CABG would suggest that almost no AMI hospitalizations in 2020 would have a CABG. These scenarios are unrealistic. While the proportion of AMI hospitalizations with a PCI are likely to continue to increase and the proportion with a CABG may continue to decrease, it is anticipated that they will plateau at some point, but our data do not allow us to estimate where this could be expected to occur, although it seems likely that it will be before 2020. Similarly, the trend of shortening average LOS may continue for some years but eventually a minimum must be reached. Thus, we have not provided projections for these variables.

Our analysis has limitations. The CIHI data record only hospitalizations and not individual episodes of patient care and it was not possible to include Quebec in the analysis. The coding of hospitalizations changed from ICD-9 to ICD-10 between 2001/02 and 2002/03 in most provinces and territories but did not occur until 2003/04 in New Brunswick and 2004/05 in Manitoba. The change in coding system included a reduction in the eight-week period after an initial AMI hospitalization in which readmission for AMI-related problems would be counted as continuing care in ICD-9 to four weeks in ICD-10. This change in coding does not explain the decrease in 2006; we are unable to ascertain a reason for this reduction, but it should be noted that, if data from only 2002/03 to 2006/07 had been used, the results of the projection in numbers of AMI hospitalizations would likely have been quite different. We considered it appropriate to use all the data available rather than selectively use them. Fiscal year was used as a linear variable, which is a traditional approach, because it was considered to be the most reliable based on the limited number of years available and the structure of the data (Figures 1a and 1b).

As previously mentioned, the projection in AMI hospitalization rates did not take account of the increasing prevalence of diabetes and obesity in the Canadian population (especially in young adults) or changes in cardiovascular drug therapy, such as increasing use of statins and anti-hypertensives. It also assumed that the treatment of cardiovascular disease and AMI in particular will remain largely the same over the next 10 years, although new clinical practices, in which physicians are able to predict the prognosis for an AMI patient with greater accuracy, could emerge that would lead to a reduction in the likelihood of hospitalization for AMI or the length of stay [25-27]. These limitations point to the need for a more comprehensive and refined national disease surveillance system in Canada with which more in depth studies would be performed.

## Conclusions

Despite continued efforts to prevent cardiovascular disease, which as evidenced by the declining rate of AMI hospitalization in older Canadians, cardiovascular disease continues to be the leading cause of death and reason for hospital admissions. Moreover, there is little indication of a radical change in cardiovascular therapy that will substantially reduce the risk of AMI, whereas it is clear that the Canadian population is aging and the prevalence of diabetes and obesity is increasing, which will significantly impact the already over-stretched Canadian healthcare system. Further advances in the prevention and treatment of cardiovascular disease are required, together with an improved nationwide surveillance system of cardiovascular events. To more completely assess the burden of heart disease, it is important not only to determine how many people are hospitalized as a result of it or die from it, but also to monitor how many live with heart disease and its impact on their quality of life.

## Competing interests

At the time of writing, the authors were all full-time employees of GlaxoSmithKline Inc.

## Authors' contributions

All authors contributed to the design of the study. NSBR and JART identified and obtained the data. NSBR wrote the first draft of the manuscript and coordinated the production of the final manuscript. RC performed and ASI supervised the statistical analyses. All authors reviewed the results of the analyses and contributed to, read and approved the final manuscript.

## Authors' information

NSBR is an epidemiologist, ASI is a senior biostatistician and health outcomes manager, and JART is Director, Specialty Care in Medical Affairs, GlaxoSmithKline Inc, Mississauga, Ontario, Canada. ASI is also an assistant professor (part-time) in the Department of Clinical Epidemiology and Biostatistics at McMaster University, Hamilton, Ontario, Canada. RC held a studentship in Medical Affairs, GlaxoSmithKline Inc at the time of the statistical analyses and has subsequently returned to her PhD studies at McMaster University.

## Appendix

### A.1. Final Poisson regression model

Log (count/population) = β_0 _+ β_1_*Year + β_2_*I(≥ 75 years) + β_3_*I(65-74 years) + β_4_*I(50-64 years) + β_5_*Female + β_6_*I(Female, ≥ 75 years) + β_7_*I(Female, 65-74 years) + β_8_*I(Female, 50-64 years) + β_9_*Year*Female + β_10_*Year*I(≥ 75 years) + β_11_*Year*I(65-74 years) + β_12_*Year* I(50-64 years), where Year = 0,...,7

Tables 3 and 4

**Table 3:** Parameter estimates in the Poisson model

Parameter	Estimate (standard error)	95% Confidence interval	P-value
≥ 75 years (β_2_)	3.61(0.07)	3.48, 3.73	< 0.01
65-74 years (β_3_)	2.98 (0.07)	2.85, 3.12	< 0.01
50-64 years (β_4_)	2.34 (0.07)	2.21, 2.47	< 0.01
Female (β_5_)	-1.39 (0.08)	-1.54, -1.23	< 0.01
Female, ≥ 75 years (β_6_)	0.98 (0.08)	0.82, 1.13	< 0.01
Female, 65-74 years (β_7_)	0.63 (0.08)	0.46, 0.80	< 0.01
Female, 50-64 years (β_8_)	0.21 (0.08)	0.04, 0.37	0.01
Year (β_1_)	0.01 (0.01)	-0.01, 0.04	0.24
Year*Female (β_9_)	-0.0005 (0.01)	-0.02, 0.02	0.95
Year* ≥ 75+ years (β_10_)	-0.05 (0.01)	-0.08, -0.02	< 0.01
Year*65-74 years (β_11_)	-0.04 (0.02)	-0.07, 0.00	0.02
Year*50-64 years (β_12_)	-0.01 (0.01)	-0.04, 0.02	0.34
Scale^†^	5.96		

Deviance = 1842.83, Pearson *χ*^2 ^= 1810.59, degrees of freedom = 51, Akaike information criterion = 96.53, Bayesian information criterion = 124.60

^† ^Scale parameter estimated by square root of Pearson's *χ*^2^/degrees of freedom

**Table 4:** Estimated baseline log rates (year 2002) and time effects for eight groups

Gender*Age group	Log (rate of hospitalization)	Intercept	Slope for fiscal year
Male, ≥ 75 years	Log (count/population) =	β_0 _+ β_2_	β_1 _+ β_10 _(-0.0329)

Male, 65-74 years		β_0 _+ β_3_	β_1 _+ β_11 _(-0.0203)

Male, 50-64 years		β_0 _+ β_4_	β_1 _+ β_12 _(0.0008)

Male, < 50 years		β_0_	β_1 _(0.0149)

Female, ≥ 75 years		β_0 _+ β_5 _+ β_2 _+ β_6_	β_1 _+ β_9 _+ β_10 _(-0.0334)

Female, 65-74 years		β_0 _+ β_5 _+ β_3 _+ β_7_	β_1 _+ β_9 _+ β_11 _(-0.0208)

Female, 50-64 years		β_0 _+ β_5 _+ β_4 _+ β_8_	β_1 _+ β_9 _+ β_12 _(0.0003)

Female, < 50 years		β_0 _+ β_5_	β_1 _+ β_9 _(0.0144)

## Pre-publication history

The pre-publication history for this paper can be accessed here:

http://www.biomedcentral.com/1471-2261/12/25/prepub

## References

[B1] Tu JV Nardi L Fang J Liu J Khalid L Johansen H the Canadian Cardiovascular Outcomes Research Team National trends in rates of death and hospital admissions related to acute myocardial infarction, heart failure and stroke, 1994-2004 Can Med Assoc J 2009 180 E118 E125 http://www.cmaj.ca/cgi/reprint/180/13/E118 10.1503/cmaj.081197 19546444 PMC2696549

[B2] Foote DK Boom, Bust and Echo: Profiting from the Demographic Shift in the 21st Century 2004 Toronto: Footwork Consulting 24 28

[B3] Lee DS Chiu M Manuel DG Tu K Wang X Austin PC Mattern MY Mitiku TF Svenson LW Putnam W Flanagan WM Tu JV the Canadian Cardiovascular Outcomes Research Team Trends in risk factors for cardiovascular disease in Canada: temporal, socio-demographic and geographic factors Can Med Assoc J 2009 181 E55 E66 http://www.cmaj.ca/cgi/reprint/181/3-4/E55 10.1503/cmaj.081629 19620271 PMC2717674

[B4] Manuel DG Leung M Nguyen K Tanuseputro P Johansen H the Canadian Cardiovascular Outcomes Research Team Burden of cardiovascular disease in Canada Can J Cardiol 2003 19 997 1004 12915926

[B5] Anand SS Yusuf S Vuksan V Devanesen S Teo KK Montague PA Kelemen L Yi C Lonn E Gerstein H Hegele RA McQueen M the SHARE investigators Differences in risk factors, atherosclerosis, and cardiovascular disease between ethnic groups in Canada: the Study of Health Assessment and Risk in Ethnic groups (SHARE) Lancet 2000 356 279 284 10.1016/S0140-6736(00)02502-2 11071182

[B6] Chiu M Austin PC Manuel DG Tu JV Comparison of cardiovascular risk profiles among ethnic groups using population health surveys between 1996 and 2007 Can Med Assoc J 2010 182 E301 E310 http://www.cmaj.ca/cgi/reprint/182/8/E301 10.1503/cmaj.091676 20403888 PMC2871219

[B7] Heart and Stroke Foundation of Canada A perfect storm of heart disease looming on our horizon 2010 Toronto: Heart and Stroke Foundation of Canada http://www.heartandstroke.com/atf/cf/{99452D8B-E7F1-4BD6-A57D-B136CE6C95BF}/Jan23_EN_ReportCard.pdf

[B8] Canadian Institute for Health Information Clinical administrative databases, June 2005: privacy impact assessment 2005 Ottawa: Canadian Institute for Health Information http://www.cihi.ca/CIHI-ext-portal/pdf/internet/CAD_PIA_FINAL_APR05_EN

[B9] Tu JV Austin PC Filate WA Johansen HL Brien SE Pilote L Alter DA the Canadian Cardiovascular Outcomes Research Team Outcomes of acute myocardial infarction in Canada Can J Cardiol 2003 19 893 901 12876609

[B10] Hall RE Tu JV the Canadian Cardiovascular Outcomes Research Team Hospitalization rates and length of stay for cardiovascular conditions in Canada, 1994 to 1999 Can J Cardiol 2003 19 1123 1131 14532937

[B11] McCullagh P Nelder JA Generalized Linear Models 1989 2 London: Chapman and Hall

[B12] Pedan A Analysis of count data using the SAS system http://www2.sas.com/proceedings/sugi26/p247-26.pdf Paper 247-26

[B13] Statistics Canada Population projections for Canada, provinces and territories, 2009 to 2036 2010 Ottawa: Minister of Industry (Statistics Canada) http://www.statcan.gc.ca/pub/91-520-x/91-520-x2010001-eng.pdf

[B14] Canadian Institute for Health Information Patient cost estimator 2010 Ottawa: Canadian Institute for Health Information http://www.cihi.ca/CIHI-ext-portal/internet/en/ApplicationNew//spending+and+health+workforce/spending/CIHI020209

[B15] Rawson NSB Malcolm E Validity of the recording of ischaemic heart disease and chronic obstructive pulmonary disease in the Saskatchewan health care datafiles Stat Med 1995 14 2627 2643 10.1002/sim.4780142404 8619104

[B16] Tu JV Austin P Naylor CD Naylor CD, Slaugher PM Acute myocardial infarction outcomes in Ontario Cardiovascular health services in Ontario: an ICES atlas 1999 Toronto: Institute for Clinical Evaluative Sciences 83 110

[B17] Austin PC Daly PA Tu JV Multicenter study of the coding accuracy of hospital discharge administrative data from patients admitted to cardiac care units in Ontario Am Heart J 2002 144 290 296 10.1067/mhj.2002.123839 12177647

[B18] Kennedy CE Brien SE Tu JV the Canadian Cardiovascular Outcomes Research Team An overview of the methods and data used in the CCORT *Canadian Cardiovascular Atlas *project Can J Cardiol 2003 19 655 663 12772015

[B19] Manuel DG Lim JJY Tanuseputro P Stukel TA How many people have had a myocardial infarction? Prevalence estimated using historical hospital data BMC Public Health 2007 7 174 http://www.biomedcentral.com/1471-2458/7/174 10.1186/1471-2458-7-174 17650341 PMC1994682

[B20] Lee DS Johansen H Gong Y Hall RE Tu JV Cox JL the Canadian Cardiovascular Outcomes Research Team Regional outcomes of heart failure in Canada Can J Cardiol 2004 20 599 607 15152289

[B21] Kapral MK Laupacis A Phillips SJ Silver FL Hill MD Fang J Richards J Tu JV the Investigators of the Registry of the Canadian Stroke Network Stroke care delivery in institutions participating in the Registry of the Canadian Stroke Network Stroke 2004 35 1756 1762 10.1161/01.STR.0000130423.50191.9f 15143293

[B22] Public Health Agency of Canada Report from the National Diabetes Surveillance System: Diabetes in Canada, 2009 2009 Ottawa: Her Majesty the Queen in Right of Canada http://www.phac-aspc.gc.ca/publicat/2009/ndssdic-snsddac-09/index-eng.php

[B23] Ko DT Tu JV Samadashvili Z Guo H Alter DA Cantor WJ Hannan EL Temporal trends in the use of percutaneous coronary intervention and coronary artery bypass surgery in New York state and Ontario Circulation 2010 121 2635 2644 10.1161/CIRCULATIONAHA.109.926881 20529997

[B24] Roe MT Messenger JC Weintraub WS Cannon CP Fonarow GC Dai D Chen AY Klein LW Masoudi FA McKay C Hewitt K Brindis RG Peterson ED Rumsfeld JS Treatments, trends, and outcomes of acute myocardial infarction and percutaneous coronary intervention J Am Coll Cardiol 2010 56 254 263 10.1016/j.jacc.2010.05.008 20633817

[B25] Kotowycz MA Pal Syal R Afzal R Natarajan MK Can we improve length of hospitalization in ST elevation myocardial infarction patients treated with primary percutaneous coronary intervention? Can J Cardiol 2009 25 585 588 10.1016/S0828-282X(09)70717-2 19812804 PMC2782502

[B26] Kotowycz MA Cosman TL Tartaglia C Afzal R Pal Syal R Natarajan MK Safety and feasibility of early hospital discharge in ST-segment elevation myocardial infarction - a prospective and randomized trial in low-risk primary percutaneous coronary intervention patients (the Safe-Depart Trial) Am Heart J 2010 159 117.e1 e6 http://www.sciencedirect.com/science?_ob=MImg&_imagekey=B6W9H-4XY3SNN-P-7&_cdi=6683&_user=110177&_pii=S0002870309008229&_origin=gateway&_coverDate=01%2F31%2F2010&_sk=998409998&view=c&wchp=dGLbVlW-zSkzS&md5=7291b5d5428c159be87f4277bf25a913&ie=/sdarticle.pdf 10.1016/j.ahj.2009.10.024 20102876

[B27] Hlatky MA Heidenreich PA The year in epidemiology, health services research, and outcomes research J Am Coll Cardiol 2011 37 1859 1866 21545941 10.1016/j.jacc.2011.01.020

